# Effects of remote ischemic conditioning on kidney injury in at-risk patients undergoing elective coronary angiography (PREPARE study): a multicenter, randomized clinical trial

**DOI:** 10.1038/s41598-019-47106-7

**Published:** 2019-08-19

**Authors:** François Roubille, Jean-Christophe Macia, Fabrice Ivanes, Denis Angoulvant, Victor Mateus, Loïc Belle, Meyer Elbaz, Olivier Morel, Alain Furber, Loïc Bière, Fabrice Prunier

**Affiliations:** 1 PhyMedExp, Université de Montpellier, INSERM, CNRS, Cardiology Department, CHU de Montpellier, Montpellier, France; 2 Université de Tours, EA 4245 Transplantation Immunité Inflammation & Loire Valley Cardiovascular Collaboration, CHRU de Tours, Service de Cardiologie, Tours, France; 3 Centre hospitalier de Laval, Service de Cardiologie, Laval, France; 4 Centre hospitalier d’Annecy, Service de Cardiologie, Annecy, France; 5 Université Toulouse, CHU Toulouse, Service de Cardiologie, Toulouse, France; 6 Université de Strasbourg, Service de Cardiologie, Strasbourg, France; 7 Institut MITOVASC, UMR INSERM U1083 and CNRS 6015, Service de Cardiologie, CHU Angers, Université Angers, Angers, France

**Keywords:** Kidney, Interventional cardiology

## Abstract

The ability of remote ischemic preconditioning (RIPC) to prevent contrast-induced nephropathy (CIN) following percutaneous coronary angiography in at-risk patients is controversial. No evidence exists regarding potential RIPC positive effects on renal function and clinical outcomes in the long-term. The PREPARE study was a randomized, prospective, multicenter, and double-blinded trial. A total of 222 patients scheduled for coronary angiography and/or percutaneous transluminal coronary angioplasty with an estimated glomerular filtration rate (eGFR) < 40 mL/min/1.73 m^2^, or eGFR between 40 and 60 mL/min/1.73 m^2^ and two further risk factors were allocated to RIPC or control groups. Preventive measures were applied to all patients, including continuous intravenous saline infusion, withdrawal of nephrotoxic drugs, and limited volume of contrast medium. The primary endpoint, namely incidence of CIN, was 3.8% in the control group and 5.1% in the RIPC group (p = 0.74). The secondary endpoints, *i.e*., changes in serum creatinine and eGFR levels from baseline to 48 hours and from baseline to 12 months following contrast medium exposure, did not differ between both groups. The incidences of all major clinical events at 12 months were similar in both groups. In this population at risk of CIN, preventive strategies were associated with low CIN incidence. RIPC impacted neither the CIN incidence nor both the renal function and clinical outcomes at 1-year follow-up.

## Introduction

Contrast-induced nephropathy (CIN) is a leading cause of hospital-acquired acute kidney injury^1^, which has been associated with significant morbidity and mortality^2^. Despite prophylactic measures like the systematic use of hydration protocols, CIN remains a significant complication after coronary angiography and percutaneous transluminal coronary angioplasty (PTCA). Defined as a relative 25% rise or 0.5 mg/dL increase in serum creatinine values compared with baseline within 48–72 hours after contrast medium administration, the CIN incidence (2–40%) highly depends on several risk factors, such as moderate-to-severe chronic renal impairment, age, diabetes mellitus, heart failure, shock, left ventricular systolic dysfunction, concomitant use of nephrotoxic drugs, large volumes of contrast agent, and anemia^3,4^. These risk factors are frequently associated in patients undergoing coronary angiography and PTCA, and new strategies are hence required to effectively prevent CIN. First assessed within the heart^5^, remote ischemic preconditioning (RIPC) is a conditioning strategy in which an organ or tissue other than the target is exposed to brief periods of ischemia-reperfusion for conditioning^6–8^. Simple and inexpensive, the RIPC technique using transient limb ischemia as a stimulus has emerged as a smart approach for a wide range of clinical scenarios including acute kidney injury^9,10^. Recent proof-of-concept trials have reported that RIPC was associated with significantly reduced CIN rates^11–13^. These encouraging results when using RIPC before coronary angiography in patients at risk of CIN still have limited clinical implication as only few patients have been tested and owing to the lack of evidence regarding its ability to reduce renal injury and its potentially associated clinical outcomes at longer-term.

The renal PRotection against contrast mEdium-induced nephropaPAthy in patients at Risk undErgoing coronary angiography (PREPARE) trial was therefore designed to determine if RIPC, when initiated before coronary angiography or PTCA, would reduce CIN occurrence in patients at risk of CIN, and to assess its potential beneficial effects at 12 months.

## Methods

### Study design

The PREPARE study was a randomized, prospective, multicenter, and double-blinded trial. The ethics committee of the Angers University Hospital approved the protocol (reference 2014/28), and the study was conducted in accordance with the Helsinki Declaration and French law. Written informed consent was provided by all participants prior to inclusion in the study. The study was registered at ClinicalTrials.gov (04/06/2015 Identifier: NCT02463604).

### Patients

Patients admitted at the French hospitals of Angers, Annecy, Laval, Montpellier, Toulouse, Tours, and Strasbourg were prospectively enrolled in the study. Inclusion criteria were: (1) patients scheduled for coronary angiography and/or percutaneous transluminal coronary angioplasty; (2) patients with an estimated glomerular filtration rate (eGFR), determined using the modification of diet in renal disease (MDRD) formula, <40 mL/min/1.73 m^2^, or between 40 and 60 mL/min/1.73 m^2^ and two further risk factors in addition among age ≥75 years, diabetes mellitus, or heart failure III or IV. Exclusion criteria were: age <18 years, no written informed consent, expected low volume of intravascular contrast medium upon routine coronary angiography prior to valvular cardiac surgery or in the dilated cardiomyopathy setting, dialysis, urgent angiography in STEMI, cardiogenic shock requiring catecholamine infusion, systolic blood pressure <80 mmHg, intra-aortic balloon counter-pulsation, contrast medium injection within the previous 30 days, impossibility to perform RIPC, as well as expected impossibility to obtain follow-up data at 1-year follow-up.

### Experimental protocol

Recommended hydration consisted of saline 0.9% solution infusion at a rate of 1 mL/Kg/h for 12 hours prior to contrast medium injection and up to 12 hours thereafter. Metformin, angiotensin-converting enzyme inhibitors, angiotensin II receptor blockers, diuretics, and non-steroidal anti-inflammatory drugs were discontinued at least 24 hours before the angiography. The patients were randomly assigned in a 1:1 ratio to either the control group or RIPC group using a minimization algorithm based on age <75 or ≥75 years, diabetes mellitus absence or presence, NYHA heart failure I/II or III/IV, hematocrit <39% or ≥39%, and eGFR < 20, 20–39 or 40–60 mL/min/1.73 m^2^. Randomization was performed by a non-blinded research nurse using a web-based system (Clinsight). The RIPC group underwent four cycles of 5-min inflation to 200 mmHg and 5-min deflation using a standard upper-arm blood-pressure cuff. The control group underwent a sham procedure similar to RIPC, *i.e*., four cycles of 5-min inflation and 5-min deflation using a standard upper-arm blood-pressure cuff, with the cuff inflated to 10 mmHg to simulate the feeling of a treatment being applied. The nurse in charge of the cuff inflation ensured that both the patient and interventional cardiologist were unable to read the inflation pressure applied. All other investigators were blinded to treatment assignment for the duration of the study, and the patients were not told which inflation would be beneficial in preventing kidney injury. The time between the end of last cuff inflation and beginning of coronary intervention was set between 5 and 60 min. Coronary angiography and PTCA procedures were conducted using standard techniques. Blood samples were drawn prior to coronary intervention and sampling was repeated at 48 hours and 1 year following contrast medium injection. An independent investigator blinded to group allocation carefully collected baseline characteristics, discharge treatments, serum creatinine levels, eGFR, and clinical events at 12 months after contrast medium exposure by telephone contact with the patient, general practitioner, and cardiologist. Major events comprised death from all causes, cardiovascular death, non-fatal infarction, hemofiltration or hemodialysis, and congestive heart failure leading to hospital admission.

### Endpoints

The primary endpoint was the CIN incidence, defined as an absolute rise of ≥44 µmol/L (0.5 mg/dL) or 25% increase in serum creatinine levels from baseline within 48 hours after contrast medium exposure.

The secondary endpoints were changes in serum creatinine and eGFR levels from baseline up to 48 hours and 12 months following contrast medium exposure, along with the incidence of major clinical events at 12 months.

### Sample size assessment

The reported CIN incidence proves highly variable, ranging from 8^14^ to 40%^11^, while highly dependent on studied patients’ risk profiles. Among 1,632 consecutive coronary angiographies or PTCA performed at the Angers university hospital in 2014, we observed a 20% CIN incidence (unpublished data). When selecting among these patients those meeting the PREPARE study criteria for high CIN risk, CIN incidence reached 28%. We hence considered a 28% CIN incidence for sample size calculation in view of the PREPARE study. RIPC has been previously associated with a 70% and 58% CIN reduction, respectively,in patients at lower risk of acute kidney injury^11,12^. For our research, sample size calculation was based on the assumption that RIPC would reduce CIN incidence by 60%. In order to reach a statistical power of 80%, and significance level of 0.05, we estimated the required sample size to be 220 patients.

### Statistical analysis

Statistical analyses were performed using SPSS 15 (SPSS, Inc. Chicago, IL, USA). The Kolmogorov-Smirnov test was used to assess the distribution assumptions for normality. Continuous variables are expressed as mean ± standard deviation (SD) or median plus inter-quartile range, where appropriate. Categorical variables were given as counts or absolute frequencies. Differences between groups were assessed using chi-squared or Fisher’s exact test for categorical data and Student t-test or Mann-Whitney test for continuous data. Endpoints were assessed using a per-protocol analysis. All analyses were two-sided, and statistical significance was defined as a p-value < 0.05.

## Results

### Study population characteristics

**Figure 1:**
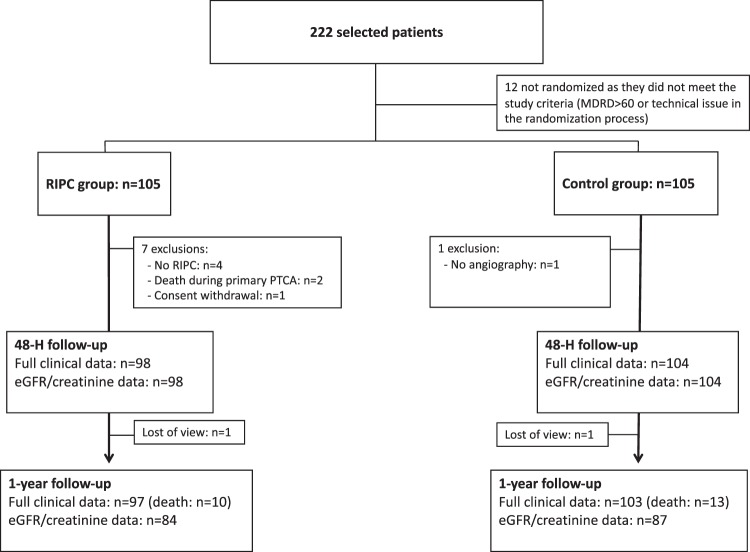
Flowchart of patients in the PREPARE study. PTCA = percutaneous transluminal coronary angioplasty; eGFR = estimated glomerular filtration rate.

A total of 222 patients were enrolled in the study, with 12 patients excluded prior to randomization (Fig. 1). Eight patients, namely seven in the RIPC group and one in the control group, were excluded from analysis after randomization. The reasons for exclusion were: RIPC procedure not performed in four cases, death at the time of angioplasty in two, coronary angiography not performed in one, and consent withdrawal in the remaining one.

Baseline characteristics of the study population have been presented in Table 1. Mean patient age was 76 ± 8 years, with 70% being male, and 52% diabetics. There were no significant differences in baseline characteristics between both groups except for the body mass index (29.4 ± 5.3 in RIPC group *vs*. 27.6 ± 5.2Kg/m^2^ in the control group, p = 0.02). PCI occurred in 46% of the patients in the control group (1 treated lesion in 75%, 2 in 21%, and 3 in 4%) and 35% in the RIPC group (1 treated lesion in 85%, 2 in 15%), p = 0.12. As shown in Table 2, baseline medications including angiotensin-converting enzyme inhibitors, angiotensin II receptor blockers, loop diuretics, spironolactone/eplerenone, metformin, and non-steroïdal anti-inflammatory drugs did not significantly differ between both groups.

**Table 1:** Baseline characteristics of the study population.

	Control group	RIPC group	p value
	(n = 104)	(n = 98)	
Age, years	76.7 ± 7.6	75.6 ± 9.2	0.33
Male, n (%)	72 (69)	68 (69)	0.55
Body-mass index, kg/m^2^	27.6 ± 5.2	29.4 ± 5.3	0.02
**CV risk factors**			
Diabetes, n (%)	53 (51)	54 (55)	0.33
Dyslipidemia, n (%)	66 (63)	70 (71)	0.15
Current smoker, n (%)	12 (12)	13 (13)	0.44
Hypertension, n (%)	91 (88)	87 (89)	0.48
**Procedure**			
Angiography/PCI, n (%)	56 (54)/48 (46)	64 (65)/34 (35)	0.12
Volume of contrast used, mL	73 [45;121]	80 [50;111]	0.57
**Clinical data**			
Systolic blood pressure, mmHg	131 ± 24	137 ± 20	0.11
Diastolic blood pressure, mmHg	71 ± 11	73 ± 11	0.11
Heart rate, beats/min	71 ± 15	72 ± 19	0.61
NYHA III/IV, %	27 (26)	27 (28)	0.63
Killip class 1/2/3, n (%)	75 (72)/26 (25)/3 (3)	80 (82)/15 (15)/3 (3)	0.22
Sinus rhythm, n (%)	73 (70)	79 (81)	0.08
**Ejection fraction**			
>49%, n (%)	38 (37)	39 (40)	
<49–40>, n (%)	9 (9)	18 (18)	
<40, n (%)	37 (35)	25 (26)	
Unknown, n (%)	20 (19)	16 (16)	0.15
Hemoglobin, g/dL	12.4 ± 1.9	12.6 ± 1.7	0.38
Hematocrit, %	37.3 ± 5.2	37.3 ± 5.2	0.36
Baseline serum creatinine, µmol/L	147 ± 44	149 ± 50	0.7
Baseline eGFR mL/min/1.73m^2^	42 ± 10	43 ± 11	0.85
**Mehran score**			
Mean	7.7 ± 3.5	8.0 ± 3.7	0.61
<5, n (%)	19 (18)	18 (18)	
<6–9>, n (%)	52 (50)	47 (48)	
<10–14>, n (%)	27 (26)	27 (28)	
>14, n (%)	6 (6)	6 (6)	0.98

Data are presented as %, and mean ± SD.

**Table 2:** Baseline medications.

	Control group (n = 104)	RIPC group (n = 98)	*P value*
ACE inhibitor, n (%)	38 (37)	36 (37)	0.76
Angiotensin II receptor blocker, n (%)	36 (35)	30 (31)	0.36
Loop diuretic, n (%)	61 (59)	62 (63)	0.88
Thiazide diuretic, n (%)	14 (13)	13 (13)	0.84
Spironolactone/eplerenone, n (%)	14 (13)	11 (11)	0.52
Metformin, n (%)	17 (16)	19 (19)	0.71
NSAID, n (%)	2 (2)	0 (0)	0.16

ACE: angiotensin converting enzyme; NSAID: non-steroïdal anti-inflammatory drugs.

### Endpoints

As shown in Table 3, the overall CIN incidence was nine out of 202 (4.5%), involving four patients in the control group (3.8%) and five in the RIPC group (5.1%; p = 0.74).

**Table 3:** Outcomes.

	Control group (n = 104)	RIPC group (n = 98)	*P value*
**Primary endpoint**			
Contrast induced nephropathy, n (%)	4 (3.8)	5 (5.1)	0.74
**Secondary endpoints**			
Change in serum creatinine from baseline to 48 hours, µmol/L	−9 [−23;9]	−5 [−16;6]	0.20
Change in eGFR from baseline to 48 hours, mL/min/1.73 m^2^	3 [−2;10]	1 [−2;5]	0.22
Serum creatinine at 12 months, µmol/L	147 ± 65	141 ± 56	0.49
eGFR at 12 months, mL/min/1.73 m^2^	46 ± 16	47 ± 15	0.58
Change in serum creatinine from baseline to 12 months, µmol/L	−5 [−23;16]	−6 [−25;10]	0.68
Change in eGFR from baseline to 12 months, µmol/L	2 [−6;12]	3 [−4;9]	0.71
One-year all-cause mortality, n (%)	13 (13)	10 (10)	0.63
One-year cardiovascular mortality, n (%)	8 (8)	8 (8)	0.90
One-year non-fatal myocardial infarction, n (%)	4 (4)	4 (4)	1.00
One-year non-fatal stroke, n (%)	1 (1)	0 (0)	1.00
One-year hospitalisation for heart failure, n (%)	17 (16)	16 (16)	0.93
One-year requirement for dialysis, n (%)	3 (3)	3 (3)	1.00

RIPC: remote ischemic preconditioning.

Changes in serum creatinine and eGFR levels from baseline to 48 hours following contrast medium exposure were not different between both groups. During the 12-month follow-up, 33 (16%) patients were hospitalized for heart failure, 23 (11%) died, mainly from cardiovascular etiologies, and six (3%) required dialysis. The incidences of all major clinical events were similar in both groups. Changes in serum creatinine and eGFR levels from baseline to 12 months in surviving patients were not significantly different.

## Discussion

In the PREPARE study, RIPC, initiated before coronary angiography or PTCA in at risk patients impacted neither the CIN incidence nor the renal function and clinical outcomes at 1-year follow-up. Of particular note, CIN incidence was markedly lower than expected in this population at risk of developing CIN, indicating a major role of standard preventive measures.

Although the mechanisms underlying CIN are multifactorial and not fully understood, it is well-admitted that renal ischemia and reactive oxygen species production are involved in its development^15^. Aside from a direct toxic effect on renal tubular cells, contrast medium infusion induces vasoconstriction responsible for renal ischemia, particularly in the medulla outer area^16^. When reperfusion occurs on account of diminished vasoconstriction, cells in this post-ischemic region produce huge amount of free oxygen radicals that contribute to apoptosis, at least in part through the opening of the mitochondrial transition pores (mPTP)^17^. It has been demonstrated that RIPC can activate several survival pathways that prevent both mPTP opening and cell death in the target organ^18^. Proof-of-concept studies initially reported RIPC to reduce the CIN incidence in patients undergoing coronary angiography^11–13^. Er *et al*. first reported that RIPC induced by four cycles of 5-minute inflation/5-minute deflation using a blood pressure cuff before an invasive coronary procedure dramatically diminished the CIN incidence from 40% to 12% in 100 patients with impaired renal function (serum creatinine >1.4 mg/dL or eGRF < 60 mL/min/1.73 m^2^)^11^. Of particular note, the 40% CIN incidence in Er’s control group was outstandingly high in this study as compared with the 4% observed in the PREPARE control group. This high CIN incidence in Er’s study occurred despite implementing preventive strategies including N-acetylcysteine pretreatment, adequate continuous intravenous saline infusion before and after angiography, withdrawal of nephrotoxic drugs before the procedure, as well as applying limited volumes of contrast medium. The study populations in both studies were similar except for diabetes incidence, which proved to be higher in Er’s study (64%) than in PREPARE (52%). While diabetes is a major risk factor of developing CIN, its interference in the protective effect of RIPC is likewise a subject of controversies^19^. In the recent EURO-CRIPS study, 223 patients with moderate renal impairment, defined by eGFR of 30–60 mL/min/1.73 m^2^, and candidates for elective PCI were randomized to receive either RIPC or sham intervention^20^. RIPC significantly reduced CIN incidence from 26% to 12%. Of note, RIPC displayed no benefits in the pre-specified subgroup of diabetics.

It must be emphasized that these two studies demonstrating positive RIPC effects reported high CIN incidences in their control groups (40% in Er’s study and 26% in the EURO-CRISP study). More recently, others authors reported RIPC to be associated with mitigated or lacking effects in patients exhibiting lower CIN incidences. In the RIPCIN study, 76 patients at risk of CIN were randomized to receive either RIPC (four cycles of 5-minute inflation/5-minute deflation using a forearm blood pressure cuff) or Sham procedure before radiological intervention with expected >100 mL contrast medium infusion^21^. CIN risk was defined with comparable parameters than those applied in the PREPARE study: eGFR < 45 mL/min/1.73 m^2^ or <60 mL/min/1.73 m^2^ with diabetes mellitus, or <60 mL/min/1.73 m^2^ and two additional risk factors. All patients received a standard hydration protocol before the procedure. The changes in serum creatinine levels from baseline to 48–72 hours after contrast medium administration were not different between RIPC and Sham groups. CIN occurred in four patients only (6%), in line with the 4% observed in PREPARE. In a subgroup of patients with a very high CIN risk (Mehran risk score ≥11), RIPC significantly prevented the increase in serum creatinine levels. The positive results in this subgroup analysis, however, require cautious interpretation due to the small patient numbers involved (five in Sham; six in RIPC). While RIPC may be protective in selected patients at very high risk of CIN, it is often difficult to clearly identify which patients would actually benefit from a RIPC strategy. Even if the majority of trials reported being able to identify at-risk population based on the Mehran’s score or factors included in that score, while applying similar preventive strategies including saline hydration prior to and after contrast medium injection, and discontinuation of nephrotoxic drugs 24 hours prior to the angiography, one major risk factor remains the volume of contrast medium administrated. For instance, the study populations in EURO-CRIPS and PREPARE were quite similar, although EURO-CRIPS patients underwent elective PCI only, whereas PREPARE patients underwent angiography and/or PCI. This was particularly relevant given that the medium volume was 174 mL in EURO-CRIPS and 97 mL in PREPARE.

In another study, Igarashi *et al*. included 60 patients with eGFR levels between 30 and 60 mL/min/1.73 m^2^ scheduled for elective angiographic^13^. In this population, very similar to the PREPARE study population, RIPC did not show any beneficial effects on CIN occurrence or eGRF evolution. Moreover, eGFR, creatinine and cystatin C levels were not significantly changed 48 hours following the angiographic procedures, suggesting low kidney toxicity in this patient population. Only the percent change in urinary liver-type fatty acid-binding protein (L-FABP) was significantly smaller in the RIPC group than in the control group, suggesting that RIPC may induce potential kidney protection. The impact of these findings on long-term renal function remains hypothetical.

The PREPARE study’s originality was to test RIPC’s potential protective effect at 48-hour after coronary angiography, along with its impact on renal function and outcomes 1 year thereafter. While previous studies were primarily focused on the first 48–72 hours, in our report, RIPC altered neither the changes in serum creatinine and eGFR levels from baseline to 12 months nor the incidence of major clinical events at 1-year in our population.

### Limits

The CIN incidence in the PREPARE study (4.5%) proved to be much lower than expected based on previous studies and on our own retrospective database (28%). This discrepancy may be partly related to the patients selected to be included in the study and, even more importantly, to the standardization of preventive measures in this randomized trial. This observation further underlines the major relevance of these preventive measures in routine practice, in an effort to limit the CIN risks. Platelet inhibitors may have interfered with RIPC, given that they have likewise been shown to exert protective effects^22^.

## Conclusion

In the PREPARE’s study population, supposed to be at risk of developing CIN, preventive strategies including continuous intravenous saline infusion before and after angiography, withdrawal of nephrotoxic drugs before the procedure, and limited volumes of contrast medium were associated with low CIN incidence. In these conditions, RIPC impacted neither the CIN incidence nor both the renal function and clinical outcomes at 1-year follow-up. Further studies are needed in populations at higher risk, specifically focused on procedures during which high volumes of contrast medium are anticipated, such as chronic total occlusion percutaneous coronary intervention.
